# Experiences of African American Breast Cancer Survivors Using Digital Scales and Activity Trackers in a Weight Gain Prevention Intervention: Qualitative Study

**DOI:** 10.2196/16059

**Published:** 2020-06-08

**Authors:** Julianne M Power, Deborah F Tate, Carmina G Valle

**Affiliations:** 1 Department of Health Behavior Gillings School of Global Public Health University of North Carolina at Chapel Hill Chapel Hill, NC United States; 2 Lineberger Comprehensive Cancer Center University of North Carolina at Chapel Hill Chapel Hill, NC United States; 3 Department of Nutrition Gillings School of Global Public Health University of North Carolina at Chapel Hill Chapel Hill, NC United States

**Keywords:** African American, cancer survivors, digital tools, weight gain prevention, qualitative

## Abstract

**Background:**

The use of digital tools to promote daily self-weighing and daily activity tracking may be a promising strategy for weight control among African American breast cancer survivors (AABCS). There have been no studies exploring the acceptability and feasibility of using digital tools for weight control or qualitative studies characterizing perceptions of daily self-weighing and daily activity tracking among AABCS.

**Objective:**

This study aimed to explore the subjective experiences of daily self-weighing and daily activity tracking using digital tools, including wireless scales and activity trackers, in a sample of AABCS participating in two technology-based weight gain prevention interventions over 6 months.

**Methods:**

Semistructured interviews (N=21) were conducted in person or over the phone, were audio recorded, and then transcribed verbatim. Each transcript was read to identify key themes and develop a codebook. Each transcript was coded using Atlas.ti software, and code outputs were used to identify overarching themes and patterns in the data.

**Results:**

On average, participants were 52.6 (SD 8.3) years of age, with obesity at baseline (BMI 33.1 kg/m^2^, SD 5.9), and weighed on 123.4 (SD 48.0) days out of the 168 days (73.5%) in the study period. Women tended to attribute their weight gain to cancer treatment and framed program benefits in terms of improved quality of life and perceptions of prolonging their survival following treatment. Using the smart scale for daily self-weighing was viewed as the tool by which participants could control their weight and improve their health and well-being posttreatment. The activity tracker increased awareness of physical activity and motivated participants to be more active.

**Conclusions:**

Participants reported positive experiences and benefits from daily self-weighing and daily activity tracking. Findings suggest that daily self-weighing and daily activity tracking using digital tools are well-received, acceptable, and feasible intervention strategies for AABCS in the context of posttreatment weight management.

## Introduction

### Background

Weight gain after breast cancer diagnosis has been associated with disease recurrence [1], increased risk of mortality [2], and other adverse and long-term effects [3]. African American women experience greater weight gain following breast cancer diagnosis [4] and worse survival outcomes compared with other breast cancer survivors [5,6]. A qualitative study found that African American breast cancer survivors (AABCS) viewed weight gain following cancer diagnosis as a significant stressor [7]. Postdiagnosis weight gain may be an important characteristic of breast cancer survivorship among African American women [7]; hence, weight gain prevention efforts should be prioritized for this high-risk population of survivors.

Although the feasibility and safety of weight control interventions have been demonstrated for breast cancer survivors [8], African American women are underrepresented in these studies [9,10]. In a recent review, only 8 weight control studies were identified specifically for AABCS [11]. Few studies have incorporated web-based or mobile technology, despite demonstrating efficacy for weight management and the potential to improve reach among racial and ethnic minority populations [12]. Only one intervention encouraged self-monitoring of weight and physical activity using digital tools [13].

Self-monitoring is a key component of behavioral weight control programs [14], and more frequent self-monitoring of body weight and physical activity has been associated with weight loss [14,15]. One possible explanation comes from social cognitive theory (SCT), which posits that individual, social, and environmental factors interact in a reciprocal manner to explain behavior [16]. According to SCT, individuals cannot self-regulate their behavior if they do not understand the conditions under which the behavior occurs or the consequences of the behavior. Therefore, successful self-regulation depends upon consistent self-monitoring done in temporal proximity to the target behavior [16]. Digital tools, such as wireless scales and activity trackers, promote adherence to self-monitoring, which has traditionally been burdensome and diminishes over time [17]. These tools allow for daily self-monitoring of weight and physical activity in the context of weight control interventions, and enable real-time data collection and delivery of tailored feedback based on objectively monitored data.

Daily self-weighing is an effective strategy for weight control that has been associated with adoption of weight control behaviors, such as reduced calorie intake and increased caloric expenditure [18]. In the only study of weight gain prevention that promoted daily self-weighing among AABCS to date, the number of self-weighing days was significantly associated with weight change [13,19], and nonadherence to daily self-weighing and daily activity tracking was associated with weight gain [19]. Concerns remain about whether frequent self-weighing might produce negative psychological consequences [20]. A recent study found no negative psychological effects of daily self-weighing in the context of a weight gain prevention intervention [21]. However, participants were young adults aged between 18 and 35 years and were predominantly white; thus, information from more diverse populations is needed [21]. The use of digital tools to promote daily self-weighing and daily activity tracking may be a promising strategy for weight control among AABCS. However, there have been no studies exploring the acceptability and feasibility of using digital tools for weight control or qualitative studies characterizing perceptions of daily self-weighing and daily activity tracking among AABCS.

### Objectives

Thus, this study aimed to explore the subjective experiences of daily self-weighing and daily activity tracking using digital tools, including wireless scales and activity trackers, in a sample of AABCS participating in the Weighing Every day for Love of Life and Body (WELL Body) program, a pilot randomized controlled trial that examined two technology-based weight gain prevention interventions for AABCS over 6 months [13]. To our knowledge, this is the first qualitative study of daily self-weighing.

## Methods

### Participants and Parent Study Design

To be eligible, participants had to be (1) female, (2) aged ≥18 years, (3) African American or black, (4) diagnosed with breast cancer in the last 10 years, (5) completed cancer treatment, and (6) showed no signs of progressive disease or a second primary cancer. Participants (N=35) were randomized to 1 of 3 groups: (1) daily self-weighing intervention (n=13); (2) daily self-weighing+ intervention (included daily activity tracking; n=11); or (3) delayed intervention control (n=11). The 6-month intervention focused on preventing weight gain via self-regulation of diet and physical activity, and daily self-weighing was promoted as the primary self-monitoring strategy. All participants were given a Bluetooth and Wi-Fi–enabled wireless scale (Withings Wireless Scale-30) [22] for use during the intervention; participants in the daily self-weighing+ intervention were also given an activity tracker (Withings Pulse) [23]. Participants had access to a website/mobile app with weight graphs and received weekly lessons and tailored feedback emails based on weight with or without activity data, depending on the intervention group. The interventions encouraged participants to view their weight information as a daily tool and indicator of progress with their diet and physical activity behaviors. The intervention took place between January 2014 and June 2015 in Chapel Hill, North Carolina. The details of the results are described elsewhere [13]. The intervention showed potential for preventing weight gain in AABCS. Median weight loss after 6 months was 0.2% in the daily self-weighing intervention and 0.9% in the daily self-weighing+ intervention versus 0.2% gain in the delayed intervention control [13]. This study was approved by the University of North Carolina (UNC) Lineberger Comprehensive Cancer Center protocol review committee and the institutional review board (IRB) of the UNC at Chapel Hill (IRB study number 13-2898). All participants provided informed consent.

### Qualitative Study Design and Data Collection

For the current qualitative study, all women randomized to the daily self-weighing or daily self-weighing+ intervention groups (n=24) were invited to participate in qualitative interviews postintervention. The qualitative subsample consisted of 21 of 24 participants who completed an interview within a month after the final 6-month assessment. Women were interviewed by 3 female PhD students and were compensated US $30. Semistructured interviews included questions focused on experiences using the digital tools provided for the intervention (Table 1). Interviews were conducted in person or by speaker phone in a private room and were audio recorded using a digital recorder. Audio recordings were transcribed verbatim and reviewed for accuracy. Demographic information was collected via baseline web-based questionnaires. Objective height and weight data were collected in person at a baseline clinic assessment.

**Table 1:** Semistructured interview questions.

Number	Questions^a^
1	*How would you describe your experience using the different technology tools provided by WELL Body^b^?*
1a	What barriers/problems, if any, did you have using the smart scale?
1b	What, if anything, would have helped you to successfully use the smart scale?
1c	What barriers/problems, if any, did you have using the app on your phone?
1d	What, if anything, would have helped you to successfully use the app?
1e	What barriers/problems, if any, did you have using the website?
1f	What, if anything, would have helped you to successfully use the website?
1g	What barriers/problems, if any, did you have using the activity tracker?
1h	What, if anything, would have helped you to successfully use the activity tracker?
2	*Before WELL Body, what other health technology tools did you use for healthy eating, weight, or physical activity?*
3	*How would you describe your commitment to using the technology tools provided by WELL Body?*
3a	Is there other information about the technology tools that you would have liked to help you in terms of how to use them to improve your diet or physical activity?
4	*Think back to the beginning of the program, how confident were you that you could successfully do the WELL Body program? On a scale of 0-10 (0=not at all confident and 10=very confident).*
4a	Were there parts of the program you were confident about that you used more than others? Describe.
5	*A key recommendation of the WELL Body program is to weigh yourself every day, and each week, your homework and feedback suggested you continue to weigh daily and use your tracker. How well do you feel you accomplished this goal?*
5a	How did you feel about weighing yourself every day?
5b	How successful were you in using the tracker every day?
6	*What activities did you do as part of the WELL Body program?*
6a	What activities/components worked best or were most helpful? How so? What ones didn’t work out well? Why?
6b	What activities/components were least helpful? How so?
6c	How did you feel about the weekly feedback? What, if anything, would have improved this portion of the program?
7	*What plans, if any, do you have for continuing to use the smart scale provided by the WELL Body program?*
8	*What benefits do you think that you, or others (eg, family), gained from your participation in WELL Body?*
8a	From using the smart scale?
8b	From using the activity tracker?
9	*How could WELL Body be re-designed to make it easier to understand/use/maintain?*
10	*What would encourage you and other African American breast cancer survivors to participate in a study like this one again?*
11	*What advice would you give to other African American breast cancer survivors considering using a smart scale?*
12	*Is there anything you would like to add about your experiences?*

^a^Italicized text represents main questions whereas nonitalicized text represents subquestions.

^b^WELL Body: Weighing Every day for Love of Life and Body.

### Qualitative Data Analysis

The primary author (JMP) performed qualitative data analysis, first reading each transcript to identify key themes and develop a codebook with deductive codes derived from the interview guide [24]. *Cancer experience* was included as an inductive code because over half of participants talked about their experience with cancer. Each transcript was coded using Atlas.ti software [25] to explore which intervention components participants talked about most frequently. For this study, the following codes were analyzed: *daily weighing*, *smart scale*, *activity tracker*, and *cancer experiences*. Code outputs were used to further explore the ideas contained within each code through descriptive analytic summaries. The analytic summaries consisted of a description of each participant’s comments related to each code. By examining the analytic summaries together, overarching themes were identified. Matrices were prepared to visualize the data and analyze patterns across participants [26]. Memo writing facilitated interpretation of the data throughout the analysis process [27]. Results from the semistructured interviews are described using illustrative quotes to highlight key findings, and participants are referred to using pseudonyms.

## Results

### Overview

On average, participants were 52.6 (SD 8.3) years of age, obese at baseline (BMI 33.1, SD 5.9), weighed on 123.4 (SD 48.0) days, and tracked physical activity on 130.3 (SD 52.4) days out of the 168 days (73.5% and 77.6%, respectively) in the study period (Table 2). There were no differences between participants who completed the interviews (n=21) and those who did not (n=3). Women tended to attribute their weight gain to cancer treatment and framed the benefits of WELL Body in terms of improved quality of life (QoL) and perceptions of prolonging their survival following cancer treatment. Several barriers and facilitators were identified to using the smart scale and activity tracker. Using the smart scale for daily self-weighing was viewed as the tool by which participants could control their weight and improve their health and well-being posttreatment. The activity tracker increased awareness of physical activity and motivated participants to be more active.

**Table 2:** Characteristics of intervention participants in WELL body (N=24).

Characteristic	Qualitative subsample (n=21)	Nonqualitative subsample (n=3)	*P* value^a^
Age (years), mean (SD)	52.62 (8.27)	51.00 (9.17)	.66
BMI (kg/m^2^), mean (SD)	33.12 (5.92)	34.51 (4.49)	.97
Years since diagnosis, mean (SD)	3.38 (2.46)	1.33 (1.16)	.12
<High school education level, n (%)	2 (9.5)	0 (0)	.07
Annual income <US $60,000, n (%)	8 (47.1)^b^	2 (66.7)	>.99
Married, n (%)	11 (52.4)	1 (33.3)	>.99
Premenopausal, n (%)	4 (19.0)	1 (33.3)	.52
Days weighed over 6 months, mean (SD)	123.38 (47.97)	89.00 (8.54)	.09
Days physical activity tracked over 6 months, mean (SD)^c^	135.30 (52.36)	80.00	.34
Percent weight change at 6 months, mean (SD)	−2.65 (5.15)	0.89 (0.77)	.11

^a^On the basis of Kruskal-Wallis tests for continuous variables and Fisher exact tests for categorical variables.

^b^n=17 (4 cases missing).

^c^Daily self-weighing+intervention group only; n=10 (qualitative subsample) and n=1 (nonqualitative subsample).

### Weight Gain Experiences During and After Cancer

One-third of participants (n=7) talked about how treatment affected their bodies, resulting in weight gain. Janet, age 57 (1 year since diagnosis), portrayed her weight gain as a process from which she felt somewhat removed. She only became aware of her weight gain after the demands of treatment were over:

For me it was only chemo, well not only chemo, but chemo and radiation and I was telling her I was sharing with friends you know I really did not notice the gain until after everything was over.

Participants described negative physical and psychological consequences of treatment, such as Carol, age 54 (2 years since diagnosis). Physical effects included limited mobility and fatigue. Psychological effects included negative body image and low self-esteem. These physical and psychological effects made it difficult for Carol to lose weight, which left her searching for a way to address her weight gain:

You hit such a low after diagnosis and treatment, and sometimes you’re not exactly healthy with the way your body looks and feels after that. You’re looking for any opportunity to, umm, make yourself feel pretty, look better, or feel better about yourself, and the weight is a big thing. So I gained a lot of weight through treatment and after, and it’s harder to get it off, so, ‘cause you can’t move like you were before and you don’t have the stamina.

Participants associated experiences of weight gain during and after cancer with a lack of control as well as negative physical and psychological consequences. Women were motivated to address weight gain resulting from treatment.

### Benefits of the Weighing Every Day for Love of Life and Body Program

Most women (n=13) contextualized their experience in WELL Body within their broader experience of cancer. A total of 5 participants framed the main program benefits in terms of enhanced recovery from treatment. Lisa, age 36 (8 years since diagnosis), viewed WELL Body as a way to deal with the weight gain and body image issues that stemmed from treatment by addressing lifestyle factors that impact overall health, improving her QoL:

Um, being a breast cancer survivor, well for me, I don’t know why I got it. Um, and I guess the best thing to do is try and live a life where you feel good, and in order to feel good, you have to be as healthy as you can… And in order to be as healthy as you can, you have to eat better, you have to exercise, so just having that sense of feeling better, and knowing that there’s a program to help you, um, feel good, and be healthy.

These ideas were reflected in the comments of other participants. WELL Body allowed Elizabeth, age 45 (5 years since diagnosis), to exert control over her weight, which she believed would decrease the likelihood of cancer recurrence and increase her chances of survival:

I am very determined to survive for as long as I can…so I want to do anything that’s going to potentially help me to do that… But I do know there are a lot of African American breast cancer survivors- whether they’re overweight or whether they’re at their ideal weight, we all struggle with the “Why?” sometimes and I think we can target a certain portion of our lives that might have an impact on whether we get it or not.

Women viewed WELL Body as a way to address weight gain resulting from treatment and control lifestyle factors that contribute to cancer risk, such as diet and physical activity. WELL Body provided an opportunity for participants to transition from treatment to survivorship by addressing long-term side effects of treatment, including weight gain.

### Barriers and Facilitators to Daily Self-Weighing

Daily self-weighing via the smart scale is an integral component of the WELL Body program. Participants described several barriers to daily self-weighing as well as corresponding facilitators, or factors that helped them overcome these barriers.

#### Initiation and Creation of a Routine

Initiating daily self-weighing was a barrier for many participants because it was viewed as an extra chore. However, creating a daily weighing routine reduced the perceived burden of daily self-weighing. Among the one-third of participants who talked about the importance of routine (n=7), the most common routine described was weighing first thing in the morning before showering. Patricia, age 48 (10 years since diagnosis), who weighed 5 days/week on average, described:

So I didn’t like the fact of starting weighing myself because it had to be a routine with me, because I had to put it into my daily morning, and you know some mornings I would forget, so, umm, as far as the weighing every day in the beginning, was a hassle but then once I put it into my routine it was good.

#### Weight Fluctuations and Increased Awareness

Another barrier to daily self-weighing was weight fluctuations. Over a third of participants (n=9) reported feeling discouraged by weight fluctuations. Helen, age 54 (1 year since diagnosis), who weighed 5 days/week on average, was very conscious of her weight and described feeling nervous about daily self-weighing when she expected her weight to be up:

Sometimes I dreaded getting on the scale, it’s like, “omigod, I’m gonna be up today.” It was a little emotional at times in just dealing with the “what is it gonna be today because I ate so and so yesterday,” to be honest with you, it was quite stressful sometimes.

Despite feeling discouraged at times, Helen reflected that by continuing to weigh daily, she became more comfortable seeing her weight fluctuate and associated these fluctuations with her diet and physical activity behaviors:

I think overall it was a positive experience because it really, especially the scale, gave me the insight into my daily routine and I am more conscious about what I eat, how much I eat, but I can say I’m truly more conscious and this study has helped me to do that… I have a better understanding of how my behavior impacted my eating habits and my activity habits, and how that really impact my daily weight.

Over half of the participants (n=14) talked about how daily self-weighing made them more aware of their diet and physical activity behaviors, which is consistent with the intervention messages participants received. Mary, age 65 (4 years since diagnosis), who weighed 6 days/week on average, described how her negative emotions toward the scale were neutralized once she realized that weight fluctuations provided her with useful information about her behaviors:

I don’t get uptight when I get on the scale anymore. That’s made a big difference and always it encourages me to weigh by just looking at it as information and just the way I look at my blood sugar…if my blood sugar goes up I don’t stop taking my blood sugar medicine, I evaluate what’s going on and I let the doctor know. So the same thing with the scale, I’m using it as a piece of health information and not, this study has really helped me not to negatively equate the scale with the negative connotation of myself.

Some participants even looked forward to stepping on the scale, such as Elizabeth, age 45 (5 years since diagnosis), who weighed 4 days/week on average. Daily self-weighing gave Elizabeth an awareness that she could control her weight. After her experience with cancer, which felt so out of her control, this realization was empowering:

I think sometimes we operate sort of in a black hole, it’s better not to know, and this forced me to know on a daily basis and so I got to the point where I really enjoyed that. I wanted to know where I was and how I could adjust those numbers and realizing that I’m completely in control for the most part, of how my weight and my BMI fluctuate. So, I think that’s what it was rooted in, just having control over a certain part of my life when most of your life often seems like you have no control over it...

#### Nonadopters

Although most participants (n=18) ultimately adopted daily self-weighing and weighed >4 days/week on average, 3 participants expressed a preference not to engage in daily self-weighing and weighed less often. Donna, age 54 (4 years since diagnosis), weighed 2 days/week on average, and described daily self-weighing as discouraging because it aroused almost a decade’s worth of frustration resulting from “out of control” feelings toward her weight. Donna’s perspective provides insight into how emotionally charged daily self-weighing can be, which is a significant barrier to adoption:

Sometimes when I’m doing exactly what I’m supposed to be doing it’s like the scale is fluctuating instead of it going [down] it’s like I’m gaining and so that frustration and just used to being at a certain weight… I feel like I’m out of control.

Daily self-weighing via the smart scale was viewed by most participants as a tool to control their weight and achieve a healthier lifestyle. However, it is important to consider how a person’s weight history might influence their desires and preferences for adopting daily self-weighing.

### Barriers and Facilitators to Daily Activity Tracking

Participants in the daily self-weighing+ intervention (n=10) group who self-monitored physical activity generally had positive feedback about their experiences using the activity tracker. Barriers included forgetting or losing the device (n=6), having problems syncing the device (n=6), and wanting more instruction on how to use the device (n=3). Linda, age 50 (2 years since diagnosis), tracked physical activity 7 days/week on average, and expressed concern about not knowing whether her device was functioning properly during the intervention:

There were times when I wasn’t sure it was working and I didn’t really understand why…sometimes I think you lose something when you have to recharge it because you have to reboot it and all this stuff. I think there were some days that it didn’t even calculate all the steps that I had walked… I think that needs a little more instruction to come with that.

In total, 2 participants talked about how the tracker worked well in conjunction with the scale to provide a comprehensive picture of their weight loss progress. Facilitators included feeling motivated by the device to reach a daily activity goal (n=7). Helen, age 54 (1 year since diagnosis), tracked her physical activity 5 days/week on average, and described how the activity tracker kept her accountable by triggering her sense of responsibility to get as much activity as possible on the days she wore the device:

I don’t know if you’ve seen the commercials where the person’s conscience is talking to him; it served as that, a little buddy on my shoulder, keeping me in line and keeping me on track of what I’m doing, and kinda forcing me to think about how to maintain and even to improve...

Sandra, age 65 (2 years since diagnosis), tracked her activity 6 days/week on average and reflected that knowing she was accumulating steps over the course of the day alleviated her worry about exercising in general and may have increased the perceived accessibility of a physically active lifestyle:

This is when I realized that your movement should be throughout the day, not just one sporadic situation where you are really working out but then the rest of the day you are just sitting around… I think it’s more moving than… worrying about exercising, but getting up and moving.

## Discussion

### Principal Findings

The purpose of this study was to explore the subjective experiences of daily self-weighing and daily activity tracking using digital tools in a sample of AABCS participating in two technology-based weight gain prevention interventions. Women viewed daily self-weighing as a way to control their weight and improve their health and well-being posttreatment. Weight fluctuations were identified as a significant barrier to daily self-weighing; however, for most, continued daily self-weighing normalized these fluctuations, allowing participants to view weight fluctuations as objective health information. The activity tracker increased awareness of physical activity and motivated participants to be more active. These findings suggest that using digital tools to self-monitor weight and physical activity is an acceptable self-regulation strategy for weight management among AABCS. Daily self-monitoring and access to real-time data may have given women a greater sense of agency and motivated them to engage in weighing and exercise behaviors.

Experiences of weight gain during and after cancer were associated with perceived lack of control. A cross-sectional study of breast cancer survivors 5 years postdiagnosis and healthy women (N=328) showed that breast cancer survivors generally perceive the world as less controllable and more random compared with healthy women; however, survivors perceive the same control over their own daily lives as healthy women [28]. This may explain why women in this study viewed their weight gain during and after cancer as a process that was largely out of their control, but were motivated to take charge of their weight by participating in WELL Body. Women viewed addressing their weight gain as a way to transition to survivorship after the illness experience. Weight gain may be viewed as a persistent symptom of cancer and its treatment [7], and continuing symptoms of cancer have been associated with psychological distress among survivors [29]. Findings from this study indicate that posttreatment weight management efforts could enhance survivorship among AABCS by increasing perceptions of personal control and reducing psychological distress related to their cancer.

One of the main benefits of the program was that participants perceived that changing their behaviors and weight would prolong their survival, which may be related to the management of uncertainty about cancer recurrence. In a study examining beliefs about breast cancer recurrence risk reduction among AABCS, more than half of the 191 respondents believed that being overweight was associated with breast cancer recurrence, and one-third agreed that losing weight could prevent recurrence [30]. A qualitative study found that breast cancer survivors were motivated to join a lifestyle intervention by fear of cancer recurrence and a desire to know they had done everything to prevent recurrence [31]. Qualitative results from a lifestyle intervention indicate that breast cancer survivors gained a sense of control over their bodies and cancer recurrence by participating [32]. In this study, frequent weight and activity information may have given participants an increased sense of control over their bodies and facilitated a perceived reduction in risk of cancer recurrence, leading to perceptions of prolonging their posttreatment survival.

In total, 3 participants expressed a preference not to engage in daily self-weighing, suggesting that daily self-weighing can be emotionally charged depending on a participant’s weight history. However, other studies have found no adverse psychological effects associated with daily self-weighing [21,33]. Gorin et al [21] found that self-weighing frequency was not associated with depressive symptoms or binge eating, but was associated with improvements in health-related QoL. Another study by Wing et al [33] found that increases in frequency of self-weighing were associated with decreases in depressive symptoms and decreases in the probability of reporting binge eating episodes per month. These studies indicate that daily self-weighing is an important aspect of weight control and may mitigate depressive symptoms and disordered eating. However, more research is needed to determine whether and to what extent daily self-weighing has any adverse psychological effects among AABCS and the larger population of breast cancer survivors as a whole.

Daily activity tracking appeared to promote awareness of current activity levels and increase motivation to be more physically active among AABCS. Another qualitative study found that daily activity tracking increased motivation to be physically active and promoted awareness around sedentary time in patients with breast cancer [34]. Taken together, these findings indicate that daily activity tracking, in conjunction with goals and advice tailored for women with breast cancer, may be an effective way to promote a more active lifestyle throughout the cancer care continuum, which could improve QoL outcomes [3]. This study identified barriers to activity tracker use, including forgetting or losing the device, which is consistent with another qualitative study [34]. Providing more detailed instructions on using the device and ensuring the availability of technical assistance may help facilitate daily activity tracking. Capitalizing on digital tools that are already integrated into participants’ everyday lives may also address barriers to daily activity tracking. These findings support the feasibility and acceptability of using activity trackers in interventions for AABCS. Future interventions promoting daily activity tracking in women with breast cancer may benefit by including messaging to remind participants to wear their trackers every day.

### Study Limitations

A key study limitation is that women voluntarily participated in the weight gain prevention interventions and may have been more motivated to address weight gain compared with other AABCS. Additionally, 3 participants did not complete qualitative interviews, so these findings may not be representative of the entire study population. Considering that 3 participants disliked and ultimately did not adopt daily self-weighing, future studies could identify different approaches to self-monitoring that are more acceptable for nonadopters. This study may have been strengthened by the inclusion of additional coders, which would have created opportunities to discuss coding disagreements and refine the coding system as well as incorporating multiple perspectives into the analytic process.

### Conclusions

This study provides a qualitative perspective of the effects of daily self-weighing and daily activity tracking in AABCS, which has previously only been explored quantitatively. Participants expressed shared positive experiences related to daily self-weighing and daily activity tracking, such as improved QoL, perceptions of prolonging their survival following cancer treatment, and greater awareness surrounding physical activity. Common barriers included difficulty initiating new habits, whereas common facilitators included creating a routine around daily self-weighing and daily activity tracking. These findings and other quantitative data [13,19] suggest that daily self-weighing and daily activity tracking are acceptable and feasible for AABCS in the context of posttreatment weight management. Daily self-weighing in this study was accompanied by health education and tailored feedback to help women make sense of weight fluctuations by relating them back to diet and physical activity behaviors. Less is known about the impact of daily self-weighing among cancer survivors in the absence of a weight control program. Additionally, few studies have explored whether daily self-weighing and daily activity tracking are useful for African American women during breast cancer treatment. Future research might explore the use of these tools during treatment to further examine the impact of daily self-weighing and daily activity tracking among African American women and to determine if preventing weight gain during treatment or posttreatment is the more optimal time for interventions aiming to improve a sense of personal control and QoL.

## Abbreviations

AABCS: African American Breast Cancer Survivors
IRB: institutional review board
QoL: quality of life
SCT: social cognitive theory
UNC: University of North Carolina
WELL Body: Weighing Every day for Love of Life and Body
